# Improvement of Component Flux Estimating Model for Pervaporation Processes

**DOI:** 10.3390/membranes10120418

**Published:** 2020-12-13

**Authors:** Botond Szilagyi, Andras Jozsef Toth

**Affiliations:** Environmental and Process Engineering Research Group, Department of Chemical and Environmental Process Engineering, Budapest University of Technology and Economics, Műegyetem rkp. 3, H-1111 Budapest, Hungary; szilagyi.botond@edu.bme.hu

**Keywords:** pervaporation modelling, model improvement, binary solution separation

## Abstract

Separating non-ideal mixtures by pervaporation (hence PV) is a competitive alternative to most traditional methods, such as distillation, which are based on the vapour–liquid equilibrium (VLE). It must be said, in many cases, accurate VLE data are already well known in the literature. They make the method of PV modelling a lot more complicated, and most of the viable models are (semi)empirical and focus on component flux (*J_i_*) estimation. The pervaporation model of Mizsey and Valentinyi, which is based on Rautenbach’s works, is further improved in this work and tested rigorously by statistical means. Until now, this type of exponential modelling was only used for alcohol–water mixtures, but in this work, it was extended to an ethyl acetate–water binary mixture as well. Furthermore, a flowchart of modelling is presented for the first time in the case of an exponential pervaporation model. The results of laboratory-scale experiments were used as the basis of the study and least squares approximation was used to compare them to the different model’s estimations. According to our results, Valentinyi’s model (Model I) and the alternative model (Model III) appear to be the best methods for PV modelling, and there is no significant difference between the models, mainly in organophilic cases. In the case of the permeation component, Model I, which better follows the exponential function, is recommended. It is important to emphasize that our research confirms that the exponential type model seems to be universally feasible for most organic–water binary mixtures. Another novelty of the work is that after PDMS and PVA-based membranes, the accuracy of the semiempirical model for the description of water flux on a PEBA-based membrane was also proved, in the organophilic case.

## 1. Introduction

Pervaporation (hence PV) is a type of membrane separation process, in which the components are separated by their different tendency to permeate through the membrane. On the feed side, the initial liquid is absorbed by the material of the polymer membrane and it is diffused through the length of the membrane, and then on the permeate side it is desorbed into the generated vacuum as a gas. In this type of process, the membrane is usually a composite membrane, which has an active and a porous supporting layer. The active layer is that which actually does the separation, by letting through the components at different degrees. The supporting layer does not take part in the separation—its only function is to provide mechanical stabilization. This is needed to counteract two forces: one is the hydrostatic pressure on the feed side, and the other one is the vacuum created on the permeate or product side [1,2].

Usually, during the separation of a binary solution, one of the components is water and the other one is an organic component. Depending on which is more likely to permeate through the membrane, there is hydrophilic (hence HPV) and organophilic pervaporation (hence OPV). There are several studies on organic–organic separation via PV, but these are not in the focus of this study [3,4,5,6].

The biggest advantage of PV, compared to the more traditional separation methods (such as distillation), is that it is not based on vapour–liquid equilibrium (hence VLE). Because of this, PV can be used to separate azeotropic solutions such as water–alcohol mixtures. So, even today, one of the most widely spread uses of PV is alcohol dehydration [7,8,9]. Further advantages are the capability of separating close-boiling point and heat-sensitive mixtures as well.

This VLE independency also poses a big problem when the aim is to model PV processes. Some of the most widely spread PV models are empirical or semiempirical [1,9], and so are heavily based on previous measurement, like in the case of the models discussed later in this work. One of best ways to characterise membrane processes is the component flux, which is the flowrate through a unit of membrane area [10]. So, it is not a surprise that most of the models focus on its estimation. Since this study focuses on the improvement of one of these models, the modelling of PV is mostly presented in the next chapter [11,12,13].

The aim of this work is to further develop a model that has been proven several times. By obtaining a better model, a more accurate calculation can support the PV process designing, via commercially used process simulation software (like ChemCAD and Aspen).

## 2. Materials and Methods

In this work, the examined aqueous mixtures can be seen in Table 1. PDMS (Sulzer PERVAP 4060), PVA (Sulzer PERVAP 1510) and, in the first case, PEBA pervaporation membranes were investigated. The standard deviation of the experimental data was 0.05. There were a minimum of three replicates in the experiments.

### 2.1. Pervaporation Modelling

Modelling of traditional separation methods such as distillation is widely researched in literature [20,21]. The research field of distillation is heavily based on the vapour–liquid equilibrium (hence VLE). On the other hand, the mechanism of membrane separation cannot be explained by VLE. One of the most basic models that can be used for membrane separation is the diffusive or pore flow model.

In this case, the driving force of the separation is the chemical potential gradient between the two sides of the membrane. Because of the constant pressure in the membrane, the chemical potential gradient can be replaced with the concentration gradient. In this way, it is easier to define one of the most important parameters of pervaporation, the membrane flux, which can be described by Fick’s first law:

(1) $$J_{i}=\frac{1}{A}\frac{dn_{i}}{dt}=-D_{i}\frac{dc_{i}}{dL}$$

Since this is a general model for most transport processes, in this context *c_i_* is the concentration outside of the membrane.

The biggest drawback of this simplistic equation is the concentration dependency of the diffusion coefficient for non-ideal mixtures. For years, the scientific community have been working on the improvement of PV models for reliable flowsheet modelling uses.

Several pervaporation models have been brought forth as viable alternatives, such as the total solvent volume fraction model, pore-flow model and solution-diffusion model [12,22,23,24]. The solution diffusion one is quite probable theory; however, there are other theories to explain the complex phenomena of this membrane process. These theories are all only hypotheses and the authors know no experiment to prove them. One of the most widely accepted explanations is the solution-diffusion model, which is applicable for two-layered composite membranes. The model can be described by the following steps [12,25]:

- absorption of components in the membrane;
- selective diffusion of components through the length of the membrane;
- desorption and consequential evaporation to vapour phase on the permeate side.

This is the model on which Rautenbach’s (1990) [25] work is based. In his work, the driving force of the chemical potential gradient is replaced by the fugacity gradient, and the diffusion coefficient is replaced by the transport coefficient. The latter change is significant because of the lesser concentration dependency of the transport coefficient [26].

Based on Fick’s equation (Equation (1)), the component flux can be described as follows:

(2) $$J_{i}=\frac{cD_{i0}}{\delta \bar{\gamma_{i}}}\left(\frac{f_{i1}-f_{i3}}{f_{i0}}\right)$$

where $\bar{\gamma_{i}}$ is the geometric mean of the activity coefficient at the two sides of the composite membrane:

(3) $$\bar{\gamma_{i}}=\sqrt{\gamma_{i1}\gamma_{i3}}$$

Introducing the transport coefficient:

(4) $$\bar{D_{i}}=\frac{cD_{i0}}{\delta }$$

Using the transport coefficient Equation (2) can be modified into:

(5) $$J_{i}=\frac{\bar{D_{i}}}{\bar{\gamma_{i}}}\left(\frac{f_{i1}-f_{i3}}{f_{i0}}\right)$$

Because the pressure at the permeate side is very low, the gas phase can be considered an ideal gas, and thus the fugacity difference can be replaced by partial pressure difference. Based on this, the partial flux can be defined as:

(6) $$J_{i}=Jy_{i}=Q_{0}\left(p_{i2}-p_{i3}\right)$$

If Equation (5) is only determined for the active layer of the composite membrane and pressure is introduced instead of fugacity, combined with Equation (6), flux can by expressed as:

(7) $$J_{i}=\frac{1}{1+\left[\frac{\bar{D_{i}}}{Q_{0}p_{i0}\bar{\gamma_{i}}}\right]}\frac{\bar{D_{i}}}{\bar{\gamma_{i}}}\left(\frac{p_{i1}-p_{i3}}{p_{i0}}\right)$$

Transport coefficient can be calculated by the following Arrhenius type equation:

(8) $$\bar{D_{i}}=\bar{D_{i}^{*}}exp\left[\frac{E_{i}}{R}\left(\frac{1}{T^{*}}-\frac{1}{T}\right)\right]$$

where T^*^ is the reference temperature, in this case equal to 293 K or 20 °C.

In the works of Mizsey and Valentinyi [26,27], Rautenbach’s equation (Equation (7)) was modified and developed into the following:

(9) $$J_{i}=\frac{1}{1+\left[\frac{\bar{D_{i}}\;\exp\left(Bx_{i1}\right)}{Q_{0}p_{i0}\bar{\gamma_{i}}}\right]}\frac{\bar{D_{i}}\;\exp\left(Bx_{i1}\right)}{\bar{\gamma_{i}}}\left(\frac{p_{i1}-p_{i3}}{p_{i0}}\right)$$

This equation is called Model I in the work of Valentinyi et al. (2013) [27] (also, the original Rautenbach model is Equation (7)). It is generally accepted that the diffusion coefficient has an intense dependence on the feed concentration. Many authors have proposed an exponential relationship between the feed concentration and diffusion coefficient, which justifies the inserting of an exponential term into the pervaporation model [10,27]. This developed model is based on empirical laboratorial data [28,29], and it is specifically recommended for polymer and composite membranes.

Furthermore, in Mizsey’s work (2005) [26], it was established that the first part of Equation (7) as well as Equation (9) can be ignored. The reasoning behind this is that the porous supporting layer’s permeability coefficient (*Q*_0_) is infinitely big compared to the transport coefficient, correlating with the concept that this layer’s resistance is negligible. Thus, the Model I can be simplified as:

(10) $$J_{i}=\frac{\bar{D_{i}}\;\exp\left(Bx_{i1}\right)}{\bar{\gamma_{i}}}\left(\frac{p_{i1}-p_{i3}}{p_{i0}}\right)$$

In this work, this equation was further improved as the following two models and researched in a similar fashion:

(11) $$J_{i}=\frac{\bar{D_{i}}\;B\;\exp\left(x_{i1}\right)}{\bar{\gamma_{i}}}\left(\frac{p_{i1}-p_{i3}}{p_{i0}}\right)$$

(12) $$J_{i}=\frac{\bar{D_{i}}\;\exp\left(x_{i1}{}^{B}\right)}{\bar{\gamma_{i}}}\left(\frac{p_{i1}-p_{i3}}{p_{i0}}\right)$$

These models are called Model II and Model III, respectively.

### 2.2. Model Improvement

As mentioned, the model research is based on empirical laboratorian data [10,14,15,16,17,18,19]. For the calculations, the following base parameters were needed:

- mole fraction of the feed (*x_i_*_1_) [mole/mole];
- mole fraction of the permeate (*x_i_*_3_) [mole/mole];
- coefficients of the Wilson equation (*A_ij_*, *A_ji_*) [cal/moleK];
- input temperature (*T*) [°C. K];
- constants of the Antoine equation for both components (*A*, *B*, *C*, *D* and *E*) [-];
- pressure on the permeate side (*p*_3_) [bar. kPa];
- partial fluxes of both components (*J_i_*) [kg/m^2^h].

The Antoine constants and Wilson parameters were obtained from the ChemCAD software’s database. Lower index numbers, as in the case of *x_i_*_1_ and *p*_3_, represent the location in the membrane module: 1 is the feed side, 2 is the intermembrane plane and 3 is the permeate side.

Based on these input parameters the following calculations can be executed. The activity coefficients can be calculated by the Wilson equation for both sides of the membrane and for both components. For example, for the component *i*, the feed side activity coefficient is:

(13) $${\ln\gamma }_{i1}=\ln\left[x_{i1}+\Lambda_{\mathrm{ij}}\cdot \left(1-x_{i1}\right)\right]+(1-x_{i1})\;\;\;\;\;\cdot [\frac{\Lambda_{\mathrm{ij}}}{x_{i1}+\Lambda_{\mathrm{ij}}\cdot \left(1-x_{i1}\right)}-\frac{\Lambda_{\mathrm{ji}}}{(1-x_{i1})+\Lambda_{\mathrm{ji}}\cdot x_{i1}}]$$

where the Λ_ij_ and Λ_ji_ coefficients were obtained by the following formulas:

(14) $$\Lambda_{\mathrm{ij}}=\frac{V_{j}}{V_{i}}\cdot \exp\left(-\frac{A_{\mathrm{ij}}}{\mathrm{RT}}\right){\;\;\;\;\Lambda }_{\mathrm{ji}}=\frac{V_{i}}{V_{j}}\cdot \exp\left(-\frac{A_{\mathrm{ji}}}{\mathrm{RT}}\right)$$

where the V_i_ and V_j_ are the molar volume of pure liquid. and it can be calculated as follows:

(15) $$V_{i}=\frac{B_{i}^{\left[1+{\left(1-\frac{T}{C_{i}}\right)}^{D_{i}}\right]}}{A_{i}}{\;V}_{j}=\frac{B_{j}^{\left[1+{\left(1-\frac{T}{C_{j}}\right)}^{D_{j}}\right]}}{A_{j}}$$

where A, B, C and D are the constants of the Antoine equation for the i and j components, respectively.

The pure component’s partial pressures can be calculated by the Antoine equation:

(16) $$p_{i0}=\exp\left(A+\frac{B}{T}+ClnT+DT^{E}\right)\cdot 10^{-5}$$

where *A*, *B*, *C*, *D* and *E* are the material depending constants of the Antoine equation.

Partial pressure in the feed and permeate side can be calculated by the Raoult’s law (Equation (17)) and Dalton’s law (Equation (18)), respectively:

(17) $$p_{i1}=p_{i0}\;x_{i1}\;\gamma_{i1}$$

(18) $$p_{i3}=y_{i3\;}\;p_{3}$$

To compare Model I, II and III, parameter fitting was used, for this purpose STATISTICA software was used. The estimated parameters were the reference transport coefficient ($\bar{D_{i}^{*}}$). component’s activation energy (*E_i_*) and the added *B* parameter of the new models. The estimated function derives from the combination of the respective model and Equation (8):

(19) $$\mathrm{Model}\;I\;\;\;\;\;J_{i}=\bar{D_{i}^{*}}\exp\left[\frac{E_{i}}{R}\left(\frac{1}{T^{*}}-\frac{1}{T}\right)\right]\left(\frac{p_{i1}-p_{i3}}{p_{i0}\;\bar{\gamma_{i}}}\right)\exp\left({B\;x}_{i1}\right)$$

(20) $$\mathrm{Model}\;\mathrm{II}\;\;\;\;\;J_{i}=\bar{D_{i}^{*}}\exp\left[\frac{E_{i}}{R}\left(\frac{1}{T^{*}}-\frac{1}{T}\right)\right]\left(\frac{p_{i1}-p_{i3}}{p_{i0}\;\bar{\gamma_{i}}}\right)B\;\exp\left(x_{i1}\right)$$

(21) $$\mathrm{Model}\;\mathrm{III}\;\;\;\;\;J_{i}=\bar{D_{i}^{*}}\exp\left[\frac{E_{i}}{R}\left(\frac{1}{T^{*}}-\frac{1}{T}\right)\right]\left(\frac{p_{i1}-p_{i3}}{p_{i0}\;\bar{\gamma_{i}}}\right)\exp\left({x_{i1}}^{B}\right)$$

As this is a nonlinear estimation process, a custom loss function needs to be defined, so least squares approximation was used. The objective function (hence OF) which needed to be minimized is the following:

(22) $$OF=\sum_{x=1}^{n}{\left(\frac{J_{i.measured}-J_{i.calculated}}{J_{i.measured}}\right)}^{2}$$

**Figure 1:**
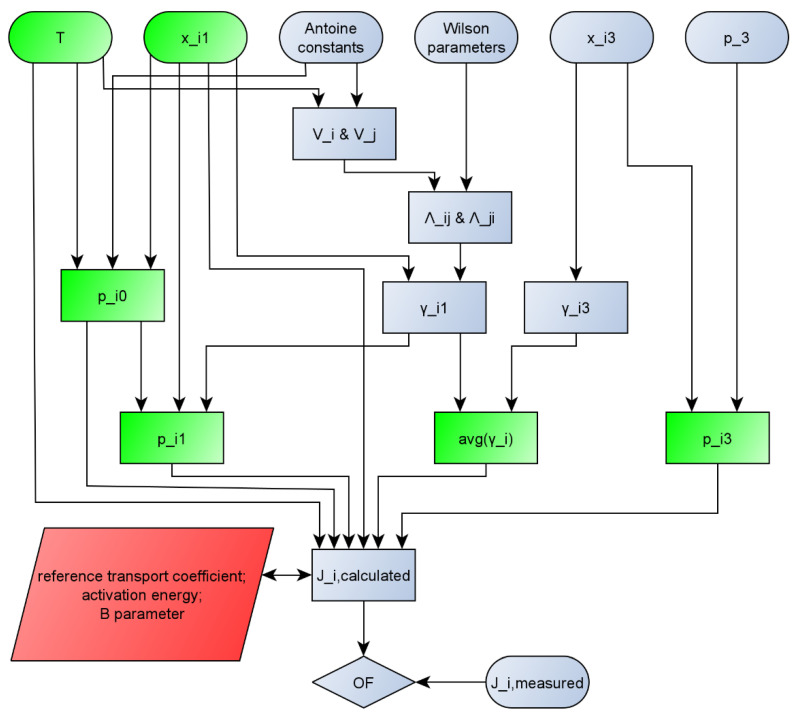
Flowchart of calculation of pervaporation modelling. (Interpretation: p_i0 means p_i0_, others can be interpreted the same way and avg(y_i) means $\bar{\gamma_{i}}$. Green parameters are the inputs of STATISTICA software. Red parameters are the parameters estimated by STATISTICA software.)

For better transparency, the calculation method that was used in this work is represented on a flowchart (Figure 1).

## 3. Results

As mentioned in the previous section, PV is mostly characterized by component flux, so during the model research it was estimated. To minimize the error, least squares approximation was used, and objective functions were obtained (Equation (22)), which can be seen in Table 2.

Based on these OFs, it can be determined which model describes real measurement most accurately. The model that has the smallest OF in any case can be considered to be more accurate.

As stated before, the examined models use three estimated parameters: the reference transport coefficient ($\bar{D_{i}^{*}}$), the component’s activation energy (*E_i_*), and the added *B* parameter. Estimation of these parameters can be seen in Table 3 for each component.

## 4. Discussion

As can be seen in Table 2., Model II gave the biggest OFs in all cases, so it did not provide us with any promise of progression. On the other hand, Model III in some cases provided even better results than Valentinyi’s Model I.

**Figure 2:**
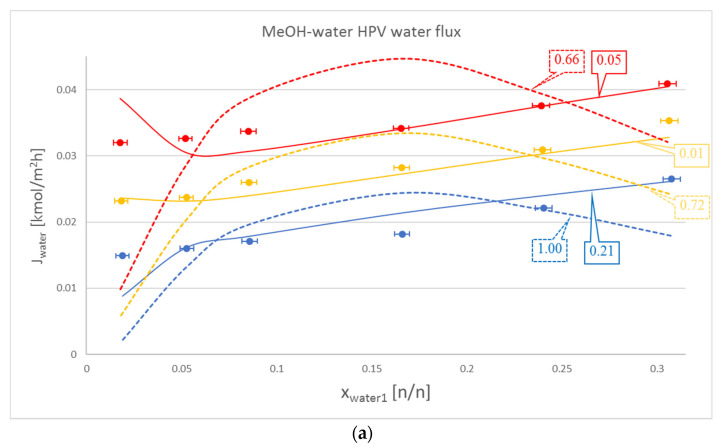

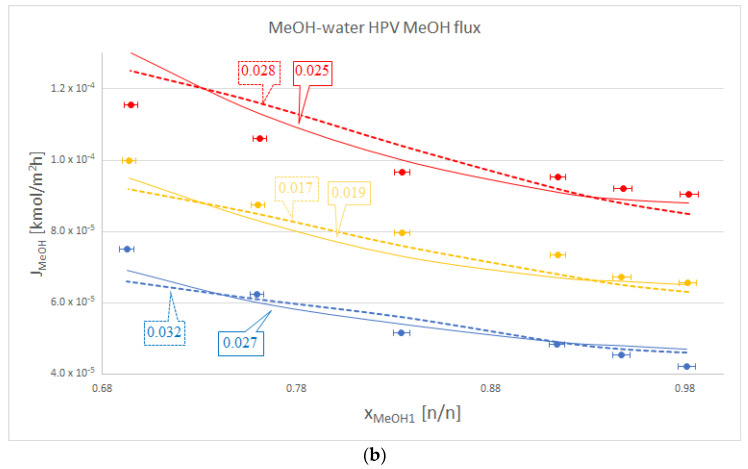
Comparison of Model I (**- - -**), Model III (**–––**) and experimental data (●), where the colour code means the following: blue: 50 °C, yellow: 60 °C and red: 70 °C. Objective functions (OFs) are represented in the text bubbles per model per temperature. (**a**) MeOH-water hydrophilic (HPV) water flux; (**b**) MeOH-water HPV MeOH flux.

In most cases, Model III proved to be better with regard to water flux modelling than Model I. These cases can be seen in Table 2 and as a representation in Figure 2a. Some cases, such as the water flux for OPV separation of isobutanol and water, are not too meaningful, but are still noticeable via OFs. The only exception is the HPV separation of isobutanol and water, where Model I beats the otherwise dominant Model III.

It is also worth mentioning that in the case of the organic component, Model III did not lag behind by much compared to Model I. This can be seen in Table 2., in the iBuOH-water and the first EtAc-water OPV results. In some cases, like both OPV and HPV separation of MeOH-water, Model III it is even better.

OFs were also analysed by temperature, so it can be determined whether the models work better at higher or lower temperature zones. OFs sorted by temperatures and mixture can be seen in Table 4. Overall, it can be said that both models behave similarly depending temperature changes and, in most cases, aqueous and organic component modelling is also indifferent. There is not a universal trend for temperature dependency, but most often higher temperatures yield lower OF. This observation needs further and bigger scale examination.

Just like in other works, our models aim to estimate the partial flux for different components, heavily based on Fick’s law (Equation (1)), and make a lot of the same assumptions and simplifications. However, the goal of most models is to find a universal linear function; meanwhile, our approach keeps the exponential expression and changes its parameter to achieve a better fit to empirical data. Another difference is that other models use the plasticisation coefficient and partial activity to estimate the locational changes of the diffusion coefficient [30], or further boundary conditions are given to achieve an Arrhenius-type equation for partial flux [31]. Some cases just use the concentration to get a linear model for liquid composition estimation in the membrane [32], while our model uses fugacity and partial pressure to achieve an exponential empirical equation. Even more complicated models use the thermodynamic functions of solubility as well as diffusion [33], while ours only uses the latter.

## 5. Conclusions

In this work, two new alternative models were proposed for component flux estimation in pervaporation processes, Model II and III. Of those two, Model II seems to be less accurate than Valentinyi’s established Model I, so no further research is worthwhile. Meanwhile, Model III seems to be better in some regards, such as in water flux estimation for OPV processes. In most cases there is little difference between Model I and III in organic flux estimation for OPV processes. Overall, it can be said that further examination is needed for the investigation of this model, but it is more than promising.

In this study, it was also determined that both Model I and III behave similarly regarding temperature changes, and at higher temperatures the models yield more realistic approximations. However, for this type of study, bigger input data are needed, so it should be researched further.

To summarise, the recommended models for the examined mixtures are listed in Table 5. The model in parentheses is also correct but less accurate. As can be mentioned, in most cases the new Model III is better in the estimation of water flux, while Model I is better for organic component flux estimation.

Model I more closely follows the nature of the exponential function, which is more compatible with the permeable target component. In conclusion, the two models describe the flux of pervaporation with sufficient accuracy. Model I is universal, while Model III can be used for the non-target component because there is no significant difference between the two models.

It must be mentioned that the exponential type model was extended to an ethyl acetate–water binary mixture, as only alcohol–water binary mixtures were examined until now. The data show that both models can work just as well for ethyl acetate as they do in the case of alcohols. So, the exponential type model most likely describes the majority of organic–water binary mixtures.

## Figures and Tables

**Table 1:** Examined mixtures.

Mixture	Type	Examined Temperatures [°C]	Water Content of Feed [wt%]	Membrane	Ref.
**OPV**					
EtOH-water	azeotropic	40, 50, 60, 70, 80	91.57–99.63	Sulzer PERVAP 4060	[14,15]
iBuOH-water	azeotropic	50, 60, 70	98.16–99.89	Sulzer PERVAP 4060	[10,16]
EtAc-water	azeotropic	50, 60, 70	98.86–99.82	Sulzer PERVAP 4060	[17]
		30, 40, 45, 50	98.93–99.80	ZSM-5 filled PEBA	[18]
**HPV**					
MeOH-water	zeotropic	50, 60, 70	1.78–3.075	Sulzer PERVAP 1510	[16,19]
iBuOH-water	azeotropic	70, 80, 90	4.57–36.39	Sulzer PERVAP 1510	[10,16]

**Table 2:** Objective functions in the case of different models.

Component	Model I	Model II	Model III
**OPV**			
water	6.0 × 10^−4^	0.003	5.7 × 10^−4^ *
EtOH	0.783 *	0.800	0.987
water	0.028	0.508	0.027 *
iBuOH	2.139 *	2.142	2.140
water	0.658	0.719	0.095 *
EtAc	0.084 *	0.087	0.086
water	1.942	5.327	1.688 *
EtAc	n/a ^1^	n/a ^1^	n/a ^1^
**HPV**			
water	2.385	6.022	0.274 *
MeOH	0.074	1.714	0.070 *
water	3.321 *	6.507	6.493
iBuOH	4.873 *	8.077	4.359 ^2^

^1^ Source did not define enough data. ^2^ Yields unrealistic physical parameter * More accurate Model.

**Table 3:** Estimated function parameters in case of the best model for each component.

Components	*E_i_* [kJ/mol]	$\bar{D_{i}^{*}}\;[\mathrm{mol}/m^{2}h]$	*B* [-]	Model
**OPV**				
water	31.28	4.94	−0.49	III
EtOH	33.09	77.78	−0.04	I
water	42.20	3.45	−22.58	III
iBuOH	−18.28	14,879.52	−1.83	I
water	30.96	6.99	−52.22	III
EtAc	8.96	8373.44	−4.48	I
water	3.69	5468.59	−0.64	III
EtAc	n/a ^1^	n/a ^1^	n/a ^1^	n/a ^1^
**HPV**				
water	23.50	167.30	−6.52	III
MeOH	30.77	0.01	−1.49	III
water	58.25	0.535	8.12	I
iBuOH	52.25	2.63	−8.06	I

^1^ Source did not define enough data.

**Table 4:** Objective functions temperature dependency.

Mixture	Temperature [°C]	Model I		Model III	
		Water	Organic	Water	Organic
**OPV**					
Water-EtOH	40	2.57 × 10^−4^	n/a ^1^	0.290	n/a ^1^
	60	1.54 × 10^−4^	n/a ^1^	0.262	n/a ^1^
	80	1.91 × 10^−4^	n/a ^1^	0.231	n/a ^1^
Water-iBuOH	50	0.011	0.011	1.973	1.995
	60	0.010	0.010	0.091	0.082
	70	0.006	0.006	0.075	0.063
Water-EtAc	50	0.159	0.007	0.052	0.056
	60	0.366	0.077	0.021	0.022
	70	0.133	0.010	0.011	0.008
Water-EtAc	30	0.302	0.121	n/a ^1^	n/a ^1^
	40	0.243	0.125	n/a ^1^	n/a ^1^
	45	0.719	0.634	n/a ^1^	n/a ^1^
	50	0.678	0.808	n/a ^1^	n/a ^1^
**HPV**					
MeOH-water	50	1.003	0.206	0.032	0.027
	60	0.720	0.013	0.017	0.020
	70	0.662	0.056	0.028	0.025
iBuOH-Water	70	1.147	2.489	1.937	n/a ^2^
	80	1.169	2.484	1.708	n/a ^2^
	90	1.004	1.490	1.203	n/a ^2^

^1^ Source did not define enough data. ^2^ Yields unrealistic physical parameter.

**Table 5:** Recommended model for examined mixtures.

Mixture	Recommended Model for	
	Aqueous Component	Organic Component
**OPV**		
EtOH-water	III (I)	I
iBuOH-water	III	I (III)
EtAc-water	III (I)	I (III)
**HPV**		
MeOH-water	III (I)	I (III)
iBuOH-water	I (III)	I

## Nomenclature

*A*	membrane area [m^2^]
*B*	constant in Model I, II and III
*c*	total molar concentration [mol/mol]
*c_i_*	concentration of component *i* [mol/m^3^]
*D_i_*	diffusion coefficient [m^2^/h]
*D*_*i*0_	diffusion coefficient of component *i* [ kmol/m^2^ h]
$\bar{D_{i}}$	transport coefficient of component *i* [kmol/m^2^ h]
${\bar{D}}_{i,exp}$	modified transport coefficient of component *i* in Model I, II and III [kmol/m^2^ h]
$\bar{D_{i}^{*}}$	relative transport coefficient of component *i* [kmol/m^2^ h]
*E_i_*	activation energy of component *i* [kJ/mol]
*f*_*i*0_	fugacity of pure *i* component [mbar, kPa]
*f*_*i*1_	fugacity of component *i* in the feed side [mbar, kPa]
*f*_*i*3_	fugacity of component *i* in the permeate side [mbar, kPa]
*J*	total flux [kg/m^2^h]
*J_i_*	partial flux [kg/m^2^h]
*L*	distance of diffusion [m]
*n_i_*	weight of component *i* [mol]
*p_i0_*	vapour pressure of pure *i* component [bar, kPa]
*p*_*i*1_	partial pressure of component *i* in the feed side [bar, kPa]
*p*_*i*2_	partial pressure of component *i* between the two layers of the membrane [bar, kPa]
*p*_*i*3_	partial pressure of component *i* in the permeate side [bar, kPa]
*p*_3_	pressure on the permeate side [bar, kPa]
*Q*_0_	permeability of the porous supporting layer of the membrane [kmol/m^2^ h bar]
*R*	gas constant [kJ/kmol K]
*t*	time [s, h]
*T*	temperature [K, °C]
*T^*^*	reference temperature: 273 K = 20 °C
*w_F_*	feed concentration of component *i* [wt%]
*x*_*i*1_	mol fraction of component *i* in the feed [mol/mol]
*y_i_*	mol fraction of component *i* in the permeate [mol/mol]
*Greek letters:*	
*β_ij_*	selectivity for component *i* and *j*
*δ*	thickness of the membrane [m]
*γ*_*i*1_	activity coefficient of component *i* in the feed
*γ*_*i*3_	activity coefficient of component *i* in the permeate
$\bar{\gamma_{i}}$	average activity coefficient of component *i*
